# Development, implementation and outcomes of a quality assurance system for the provision of continuous renal replacement therapy in the intensive care unit

**DOI:** 10.1038/s41598-020-76785-w

**Published:** 2020-11-26

**Authors:** Eloy F. Ruiz, Victor M. Ortiz-Soriano, Monica Talbott, Bryan A. Klein, Melissa L. Thompson Bastin, Kirby P. Mayer, Emily B. Price, Robert Dorfman, Brandi N. Adams, Lisa Fryman, Javier A. Neyra, The University of Kentucky CRRT Quality Assurance Group, Madona Elias, Mark Taylor, Josh McHatton, Juan Carlos Aycinena, Angel M. Diaz, Stacy A. Mason, Megan A. Perkins, B. Peter Sawaya, Kelly R. Fedder, Amanda Hornsby, Natalie Noell, Thomas A. Tribble, Jillian M. Kouns

**Affiliations:** 1 Division of Nephrology, Bone and Mineral Metabolism, Department of Internal Medicine, University of Kentucky, Lexington, KY USA; 2 College of Pharmacy, Department of Pharmacy Practice and Science, University of Kentucky, Lexington, KY USA; 3 Department of Physical Therapy, University of Kentucky, Lexington, KY USA

**Keywords:** Renal replacement therapy, Continuous renal replacement therapy, Kidney

## Abstract

Critically ill patients with requirement of continuous renal replacement therapy (CRRT) represent a growing intensive care unit (ICU) population. Optimal CRRT delivery demands continuous communication between stakeholders, iterative adjustment of therapy, and quality assurance systems. This Quality Improvement (QI) study reports the development, implementation and outcomes of a quality assurance system to support the provision of CRRT in the ICU. This study was carried out at the University of Kentucky Medical Center between September 2016 and June 2019. We implemented a quality assurance system using a step-wise approach based on the (a) assembly of a multidisciplinary team, (b) standardization of the CRRT protocol, (c) creation of electronic CRRT flowsheets, (d) selection, monitoring and reporting of quality metrics of CRRT deliverables, and (e) enhancement of education. We examined 34-month data comprising 1185 adult patients on CRRT (~ 7420 patient-days of CRRT) and tracked selected QI outcomes/metrics of CRRT delivery. As a result of the QI interventions, we increased the number of multidisciplinary experts in the CRRT team and ensured a continuum of education to health care professionals. We maximized to 100% the use of continuous veno-venous hemodiafiltration and doubled the percentage of patients using regional citrate anticoagulation. The delivered CRRT effluent dose (~ 30 ml/kg/h) and the delivered/prescribed effluent dose ratio (~ 0.89) remained stable within the study period. The average filter life increased from 26 to 31 h (p = 0.020), reducing the mean utilization of filters per patient from 3.56 to 2.67 (p = 0.054) despite similar CRRT duration and mortality rates. The number of CRRT access alarms per treatment day was reduced by 43%. The improvement in filter utilization translated into ~ 20,000 USD gross savings in filter cost per 100-patient receiving CRRT. We satisfactorily developed and implemented a quality assurance system for the provision of CRRT in the ICU that enabled sustainable tracking of CRRT deliverables and reduced filter resource utilization at our institution.

## Introduction

Continuous renal replacement therapy (CRRT) is the most common modality of renal replacement therapy (RRT) utilized for managing critically ill patients with acute kidney injury (AKI) or end-stage kidney disease (ESKD) who are hemodynamically unstable and with significant electrolyte/acid–base abnormalities or volume overload^1–3^. Although only 6–10% of all patients in the intensive care unit (ICU) require acute RRT^4–6^, mortality in these patients is high (50–60%)^3,4,7^. Thus, this is a vulnerable ICU population with a continuous need for an organized approach to specialized care.

Regardless of substantial development in the technology and delivery of CRRT to critically ill patients, for example, standardizing delivered average effluent flow rate to 20–25 ml/kg/h^8^, several aspects of CRRT delivery are not fully standardized or do not have solid evidence-based foundations^9^. Outstanding questions related to provision of CRRT remain unanswered: patient selection, timing of CRRT initiation and discontinuation, volume management, anticoagulation and the role of high-volume hemofiltration and hemoadsorption^1,3^. These factors hinder standardized provision of CRRT and result in wide heterogeneity of practice and in some cases, suboptimal care for patients^10,11^. Current research focuses on addressing these uncertainties, however, information about the delivery process and quality of CRRT is still scarce^12^.

Optimal CRRT delivery demands continuous coordination and communication among multiple stakeholders, iterative assessment and adjustment of therapy, and quality assurance systems^11,13^. A recent systematic review identified potential quality indicators for CRRT classified into three categories: structure, process and outcome^10^, which included having a specialized care team, estimating the delivered and prescribed CRRT dose, measuring the average filter life span, evaluating patient prognosis, among others^2,10,13,14^.

The success of a CRRT quality assurance system depends on team work dynamics and the selection of adequate CRRT metrics which can be captured longitudinally and monitored systematically to identify problems and generate opportunities for sustainable process improvement^11,13,15^. In this quality improvement (QI) study, we report the development, implementation and outcomes of a quality assurance system to support the provision of CRRT to adult patients in the ICU.

## Results

### Patient characteristics

We examined our 34-month experience comprising 1185 adult patients on CRRT (~ 7420 patient-days of CRRT). As shown in Table 1, overall demographic and clinical data before and after QI interventions were comparable. There were no differences in 12 out of 13 clinical characteristics reflecting demographics, AKI status or acuity of illness parameters (e.g. SOFA score). The Charlson Comorbidity Index (CCI) was slightly lower in patients examined after the QI interventions (4.0 vs. 5.0 before the QI interventions, p = 0.030).

**Table 1:** Patient characteristics before and after implementation of CRRT quality improvement interventions.

Characteristics	Total	Before QI interventions	After QI interventions	p-value^a^
**Total patients, n (%)**	1185	483	702	0.212
AKI	986 (83.2)	394 (81.6)	592 (84.3)	
ESKD	199 (16.8)	89 (18.4)	110 (15.7)	
Age (years), mean ± SD	56.6 ± 14.2	55.9 ± 13.9	57.1 ± 14.4	0.147
Sex, male, n (%)	712 (60.1)	290 (60.0)	422 (60.1)	0.980
**Race, n (%)**				0.254
White	1087 (91.7)	441 (91.3)	646 (92.0)	
Black	91 (7.7)	41 (8.5)	50 (7.1)	
Other	7 (0.6)	1 (0.2)	6 (0.9)	
Weight (kg), median [IQR]	90.9 [75.0–109.9]	90.8 [71.0–110.0]	91.0 [77.0–109.1]	0.229
Hospital LOS (days), median [IQR]	14.6 [5.7–28.8]	14.0 [6.0–27.9]	15.1 [5.4–29.4]	0.544
ICU LOS (days), median [IQR]	8.9 [3.8–19.2]	8.6 [3.8–15.5]	9.6 [3.9–20.7]	0.072
Mechanical ventilation (days), median [IQR]	4.0 [1.0–8.0]	3.5 [1.0–7.3]	4.0 [1.0–8.0]	0.278
Total CRRT days, median [IQR]	3.1 [1.4–7.0]	3.0 [1.2–6.5]	3.3 [1.6–7.4]	0.086
SOFA score at ICU admission, median [IQR]	12.0 [9.0–14.0]	12.0 [10.0–15.0]	12.0 [9.0–14.0]	0.198
SOFA score at CRRT initiation, median [IQR]	14.0 [11.0–16.0]	13.0 [11.0–15.0]	14.0 [11.0–16.0]	0.476
CCI score, median [IQR]	4.0 [2.0–7.0]	5.0 [3.0–7.0]	4.0 [2.0–6.0]	0.030
**Discharge disposition, n (%)**				0.167
Alive	507 (42.8)	219 (45.3)	288 (41.0)	
Dead	678 (57.2)	264 (54.7)	414 (59.0)	

Before QI interventions period included data from September 2016 to December 2017 (total of 16 months before and during QI interventions). After QI interventions period included data from January 2018 to June 2019 (18 months).

*AKI* acute kidney injury, *CCI* Charlson comorbidity index, *CRRT* continuous renal replacement therapy, *ESKD* end-stage kidney disease, *ICU* intensive care unit, *LOS* length of stay, *QI* quality improvement, *SOFA* sequential organ failure assessment.

^a^p-value of comparison for data before and after QI interventions.

### Quality improvement metrics

Data related to the selected CRRT QI metrics before and after QI interventions are shown in Table 2. To better visualize the trajectory of these metrics after the QI interventions, the 18-month data following QI interventions were subdivided into three 6-month periods.

**Table 2:** Selected CRRT metrics before and after implementation of CRRT quality improvement interventions.

CRRT QI metrics	Before QI interventions	After QI interventions			p-value^a^
		Jan–Jun 2018	Jul–Dec 2018	Jan–Jun 2019	
CRRT modality (CVVHDF), %	92.4%	95.1%	96.6%	100.0%	< 0.001
Anticoagulation (RCA), %	No data	23.1%	24.7%	39.5%	< 0.001
Total RCA/RCA-CRRT hours, mean ± SD	No data	0.62 ± 0.30	0.68 ± 0.27	0.73 ± 0.26	0.004
Delivered effluent dose (ml/kg/h), mean ± SD	30.50 ± 4.18	27.67 ± 2.07	28.17 ± 1.83	30.33 ± 3.14	0.939
Delivered/prescribed effluent dose, mean ± SD	0.88 ± 0.07	0.88 ± 0.02	0.88 ± 0.01	0.90 ± 0.02	0.487
Filter life span (hours), mean ± SD	26.00 ± 3.16	30.17 ± 4.96	31.00 ± 2.83	31.17 ± 3.31	0.020
Filters per patient, mean ± SD	3.56 ± 0.78	2.90 ± 0.87	2.75 ± 0.50	2.67 ± 0.64	0.054
CRRT access alarms per treatment day, mean ± SD	2.95 ± 1.02	2.02 ± 0.64	1.63 ± 0.20	1.68 ± 0.50	0.021
Total filter cost per 100-patient (USD) ± SD	80,010 ± 17,519	65,173 ± 19,614	61,744 ± 11,287	59,876 ± 14,292	0.054

The period before QI interventions included data from September 2016 to February 2017 (6 months).

*CRRT* continuous renal replacement therapy, *CVVHDF* continuous veno-venous hemodiafiltration, *RCA* regional citrate anticoagulation, *USD* United States dollars.

^a^p-value of comparison for data before QI interventions and from the last 6 months after QI interventions (Jan 2019–Jun 2019). If data before QI interventions were not available, data from the first 6 months after QI interventions (Jan 2018–Jun 2018) were used as reference.

### Quality domain: structure

Regarding the provider subdomain, we increased our initial CRRT team from 1 clinician champion, 1 nurse educator and 77 CRRT Super Users (ratio of Super Users to total ICU nurses: 0.13) in 2017 to a specialized multidisciplinary CRRT team constituted by 4 clinician champions (2 nephrologists and 2 intensivists), 3 nurse educators, 1 CRRT QI officer, 130 CRRT Super Users (ratio of Super Users to total ICU nurses: 0.21), 2 dieticians, 2 physical therapists, 1 pharmacist, and 2 bioinformaticians (Supplementary Fig. S1). Likewise, we provided instruction to our ICU nurses and clinicians to achieve a continuum of education and training as detailed in Table 3.

**Table 3:** Summary of the three CRRT quality improvement intervention phases.

**Phase I: Team development and protocol standardization (March 2017–May 2017)**	
(a) Assembly of a multidisciplinary team	Nephrologists, intensivists, ICU nurses, pharmacists, dieticians, physical therapists, technicians, bioinformaticians, ICU managers, supply chain, management and administration personnel
(b) Standardization of the CRRT protocol tailoring institutional logistics and needs	CVVHDF modality, RCA protocol (anticoagulant citrate dextrose form A), customized order set (prescription entry) in the EHR; use of a non-tunneled temporary dialysis catheter (15–20 cm long, 12–13 French) in the right internal jugular as the preferred CRRT vascular access site
**Phase II: Systematic tracking of CRRT deliverables (June 2017–September 2017)**	
(c) Creation of electronic CRRT flowsheets	Automated data extraction from the intakes and outputs flowsheet, automated transfer of machine data (e.g. fluid removal, machine pressures) and embedded calculations for suggested hourly fluid removal according to prescription
(d) Selection, monitoring and reporting of CRRT QI metrics	Ten QI metrics under 2 domains (structure and process) and 3 subdomains (provider, prescription and performance). Economic savings was also included as a QI metric
**Phase III: Training and teaching (October 2017–December 2017)**	
(e) Enhancement of education to clinicians and ICU nurses	*ICU nurse education* New user education (eighteen 4-h sessions per year) on CRRT prescription, protocols and technical aspects of the machine including circuit and filter setup, alarms management, electronic CRRT charting, among others Super user education (six 5-h sessions per year) on CRRT deliverables and in-depth review of the CRRT machine, protocols and QI activities Validator education (twelve 1-h sessions per year) on skills to verify CRRT competency of other ICU nurses *Clinician education* Tailored for residents, fellows and Faculty. Two introductory sessions and four advanced sessions per year

*CRRT* continuous renal replacement therapy, *CVVHDF* continuous veno-venous hemodiafiltration, *EHR* electronic health records, *ICU* intensive care unit, *QI* quality improvement, *RCA* regional citrate anticoagulation.

In relation to the prescription subdomain, we improved adherence to the use of continuous veno-venous hemodiafiltration (CVVHDF) from 92.4% to 100% (p < 0.001). The percentage of patients using regional citrate anticoagulation (RCA) also significantly increased from 23.1% (January–June 2018) to 39.5% (January–June 2019) (p < 0.001); and from those patients, the average ratio of the hours a patient had RCA by the total hours the same patient was on CRRT also increased from 0.62 to 0.73 (p = 0.004). There were no data pertaining to the period before QI interventions for these two RCA-related QI metrics (Table 2). It is important to note that in our program we commonly use RCA vs. no anticoagulation for CRRT, and we seldom use systemic heparin unless the patient has other specific indications for systemic anticoagulation (e.g., venous thromboembolism).

### Quality domain: process

**Figure 1:**
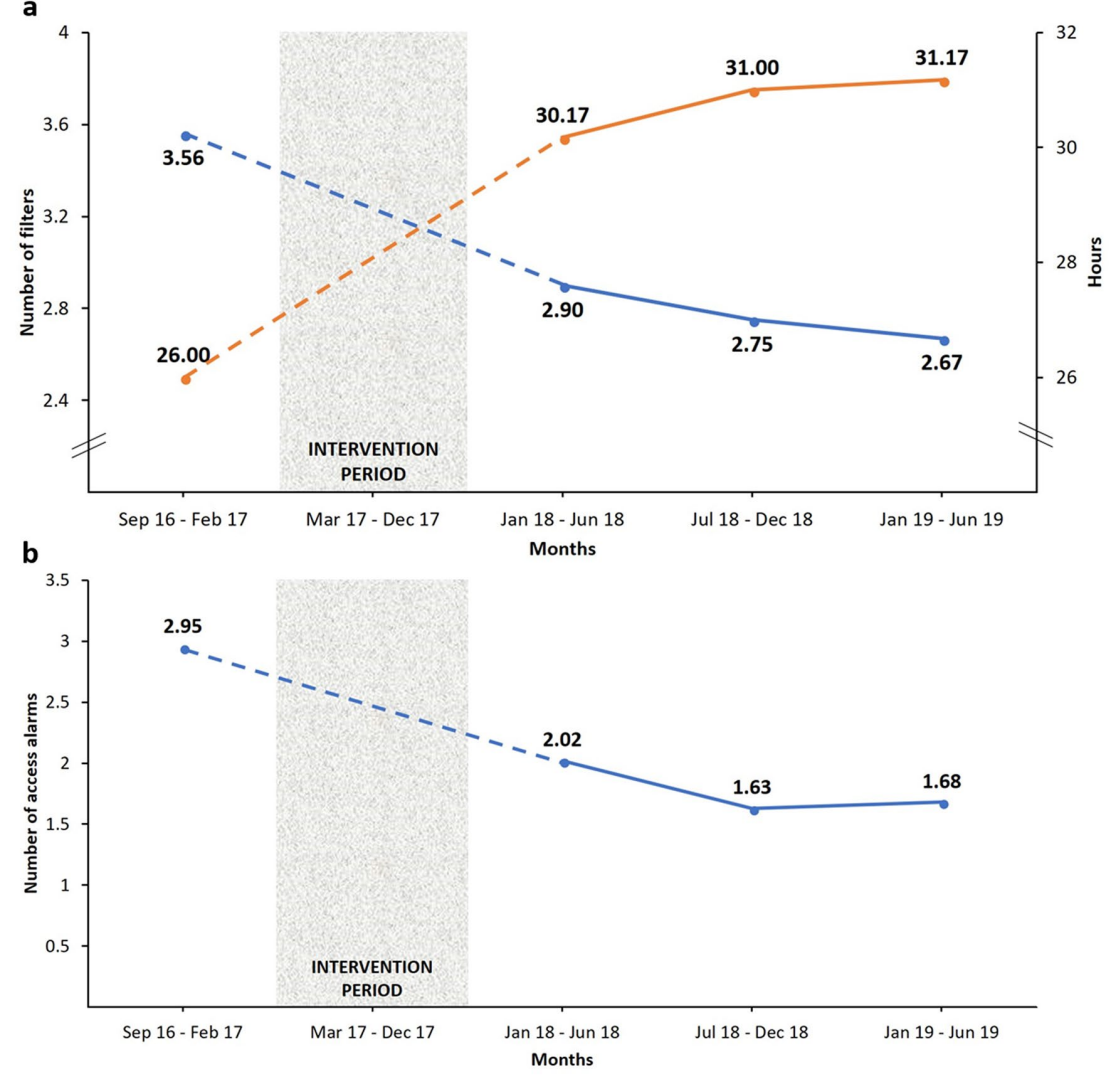
Selected CRRT performance metrics before and after quality improvement interventions: (**a**) mean number of filters used per patient (blue) and mean total hours of filter life (orange); (**b**) mean number of CRRT access alarms per treatment day. *CRRT* continuous renal replacement therapy.

When assessing the performance subdomain, the delivered CRRT effluent dose (ml/kg/h) remained stable (30.50 vs. 30.33; p = 0.939) as well as the ratio of delivered/prescribed CRRT effluent dose (0.88 vs. 0.90; p = 0.487) (Table 2). The average filter life increased from 26 to 31 h (p = 0.020), reducing the mean utilization of filters per patient from 3.56 to 2.67 (p = 0.054) (Fig. 1 and Table 2) despite similar CRRT duration (median 3.0 vs. 3.2, p = 0.194) and mortality rates (54.7% vs 56.7%, p = 0.612) when comparing the periods before QI interventions and the last 6 months post-intervention (January–June 2019). Also, the number of access alarms per treatment day was reduced by 43% (p = 0.021) (Fig. 1, Table 2).

### Economic savings

**Figure 2:**
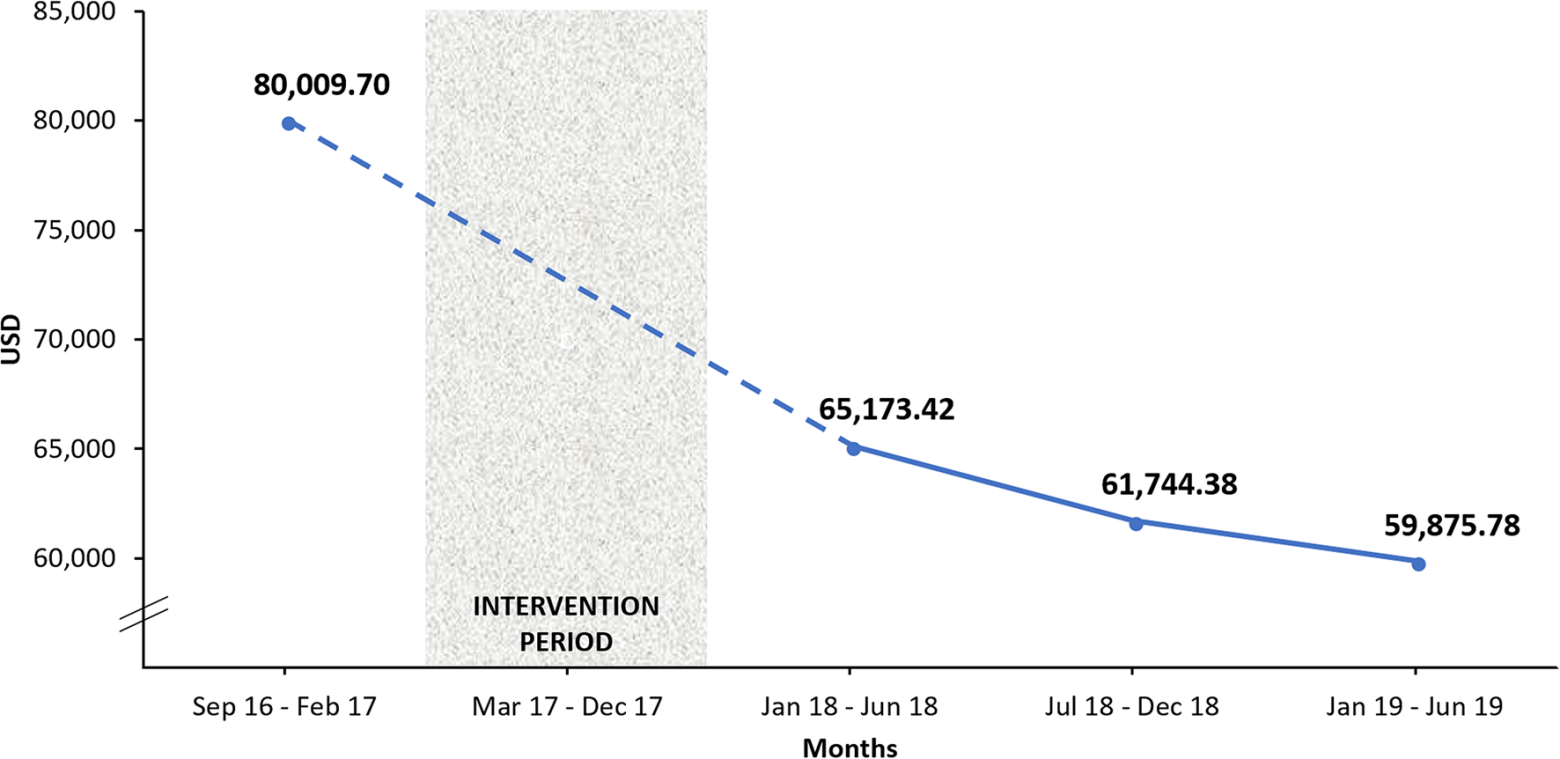
Gross filter cost per 100-patient receiving CRRT before and after quality improvement interventions.

The improvement in filter utilization translated into ~ 20,000 USD gross savings in filter cost per 100-patient receiving CRRT (p = 0.054) (Table 2, Fig. 2).

## Discussion

Critically ill patients requiring CRRT should receive their treatment in a safe, consistent and high-quality manner^16^. Previous QI studies in the CRRT population have focused on the formation of a specialized CRRT team^17,18^ and the creation of educational programs^19–21^ to improve the provision of CRRT in the ICU. Others have conducted interventions to optimize specific CRRT deliverables such as the delivered effluent dose^12,17,22^, the achievement of daily fluid removal goals^12^, as well as increasing filter life^12,20,21^, or decreasing unplanned filter changes^12^ and total CRRT downtime^17,18^. Our group has also reported QI interventions for patients on CRRT such as early rehabilitation and physical activity^23^ and the management of severe hyponatremia with customized solutions^24^. In this manuscript, we report the development, implementation and outcomes of a quality assurance system to support the provision of CRRT in the ICU at our institution. It is important to highlight that patient demographics and clinical parameters before and after the QI interventions were comparable with the only exception of the CCI score. Further, preconditions such as type of CRRT machine (Prismaflex), filter (HF1400) and catheter (Trialysis) remained unchanged throughout the study period, allowing the comparison of the selected CRRT metrics before and after QI interventions. Our report adheres to the Standards for Quality Improvement Reporting Excellence (SQUIRE 2.0) guidelines and recommendations^25^.

The QI intervention lasted 10 months and was carried out in a step-wise fashion. Several key elements to its successful implementation should be noted. First, the robust multidisciplinary collaboration between nephrologists, intensivists, ICU nurses, pharmacists and other healthcare professionals provided unique perspectives, expertise, and helped to achieve a common mindset for the provision of CRRT in the ICU (Supplementary Fig. S1). Second, the standardization of the CRRT protocol on the use of CVVHDF and RCA helped to reduce variability of CRRT delivery and maximize the providers’ competence as education was focused on a specific order set. Additionally, well-defined catheter characteristics and CRRT vascular access site helped to acquire dexterity on its handling and functionality. Third, the creation of the electronic CRRT flowsheets allowed for automated machine data transfer and embedded calculations supporting data entry for ICU nurses. Furthermore, as this tool is available on-line within the electronic health record (EHR), the clinical and QI teams were able to track CRRT delivery at any given moment. Fourth, the selection, monitoring and reporting of specific CRRT QI metrics facilitated the assessment of interim results, identification of areas for improvement, and modifications or reinforcement of good practices for better outcomes. Lastly, the establishment of a dedicated CRRT education team made it possible to continuously train and improve the workforce’s knowledge and skills. Of note, some QI metrics exhibited an early trend of continuous improvement while others only showed a significant improvement by the last 6 months post-implementation (e.g., use of RCA anticoagulation). The latter reflects real-world hindrances in CRRT delivery, which require a sustainable process of QI monitoring, continuous education, and tailored interventions to achieve the desired QI goals.

By the end of June 2019, we increased the number of multidisciplinary experts comprising the CRRT QI team and ensured a continuum of education to healthcare professionals involved in CRRT delivery. This was a major milestone because every CRRT program requires a diverse, strong and engaged core responsible for the provision of high-quality and cost-effective CRRT^11,26,27^. Some institutions have described weak collaboration between stakeholders (e.g. nephrologists and intensivists), not enough training for their healthcare professionals or lack of CRRT QI initiatives^27^. Fortunately, we were able to develop a specialized CRRT team in addition to dedicated classes for ICU nurses and clinicians held many times per year (Table 3), plus approachable educators to solve questions and concerns regarding CRRT delivery in a timely manner.

Regarding CRRT modality and anticoagulation, we were able to improve consistency of care and reduce CRRT variability by standardizing the CRRT protocol according to our local logistics and expertise. We achieved 100% CVVHDF use, ~ 40% RCA use with > 70% of the CRRT time on RCA by the end of the study period. No other form of anticoagulation is routinely used when performing CRRT in our program unless the patient has specific indications for systemic anticoagulation. Even though we increased the CVVHDF use to the maximum, adherence to the citrate protocol should be further improved as evolving evidence suggests prolonged filter life span with RCA use^28–31^. Challenges to widespread implementation of RCA use may be related to patient-specific factors (e.g., impaired liver metabolism under certain shock scenarios), feasibility of protocols for citrate titration and calcium supplementation, and clinician and nursing staff logistics and training. Furthermore, one should note that there is no evidence to support any specific CRRT modality over the other but reducing practice variability may prevent operator-related errors and therefore we selected a single modality of CRRT (e.g., CVVHDF) for our program.

CRRT dose is a dynamic metric that must be adjusted to the changing clinical needs of the patient^9^. According to Kidney Disease Improving Global Outcomes (KDIGO), it is recommended to achieve a delivered average CRRT effluent flow rate of 20–25 ml/kg/h, but ~ 30 ml/kg/h could be prescribed as interruptions, the use of pre-filter solutions and reductions in membrane permeability decrease the delivered dose^8^. Furthermore, the Acute Disease Quality Initiative (ADQI) group stated the average ratio of the effective delivered effluent dose relative to prescribed dose should be > 0.80^32^. Our results showed that the delivered CRRT effluent dose (~ 30 ml/kg/h) and the delivered/prescribed effluent dose ratio (~ 0.89) did not significantly change after QI interventions and remain adherent to guideline recommendations.

In relation to filter life, we managed to increase it from 26 to 31 h and decrease mean utilization of filters per patient from 3.56 to 2.67 despite no change in CRRT duration or mortality rates. Current evidence suggests optimization of circuit patency and filter performance primarily depends on anticoagulation practices (favoring RCA use)^30,31^, but there are still insufficient data to determine if preconditions (filter, vascular access site, catheter type) or patient characteristics (mechanical ventilation, SOFA score, calcium levels, platelet count, red blood cell transfusion, fibrinogen) may alter filter life^33^. Likewise, education to ICU nurses about filter management appears to increase its life span^20,21^, but evidence is still limited and more research is needed^33^. Therefore, it is important to document reasons for filter change and evaluate filter life span accordingly. As described by Mottes et al., there are planned filter changes (e.g., filter expiration, decision to stop therapy) and unplanned filter changes (e.g., cardiac arrest, emergent test or procedure, clotted circuit)^20^. We are currently working to improve our data collection to better examine specific reasons of filter change.

With respect to the number of access alarms per treatment, we reduced them by 43%. Although we did not assess for correlation with access placement site due to data availability, we hypothesize this could be a result of the iterative instruction to clinicians on proper catheter election and placement, in addition to enhanced education for ICU nurses on access alarm recognition, management and resolution.

The cost of CRRT relies on ICU nurse staffing salary, dialysate and/or replacement fluids, anticoagulation and extracorporeal circuit (including filter) costs^34^. It was estimated that a 24-h CVVHDF treatment costs ~ $1060 USD (excluding anticoagulation), but prices may vary from center to center^35^. After the described CRRT QI interventions, we improved our resource utilization (average filter life span and average total number of filters used per patient) and rendered ~ 20,000 USD gross savings in filter cost per 100-patient receiving CRRT at our institution. It is important to emphasize that these savings are only related to filter cost and does not account for other CRRT-related costs such as fluids, anticoagulation, monitoring or staffing salary.

Our study has some limitations to consider when interpreting the results. First, given the multifaceted quality assurance implementation, we cannot prove which specific QI intervention impacted any given outcome (e.g., RCA use vs. nurse education for improvement in filter life). Further, residual confounding is possible given the observational nature of this QI study. Second, this work was primarily planned to improve CRRT local care according to the characteristics and logistics of our institution, therefore our approach may not be applicable to other centers. Likewise, additional monitoring may be necessary to evaluate the sustainability of these results over time. However, we established a framework for quality assurance, analyzed a large number of patients (~ 7420 patient-day of CRRT) and made several measurements of CRRT QI metrics accepted in our scientific community to compare outcomes before and after QI interventions. Future directions include expanding our data collection to have additional tracking of CRRT QI metrics (e.g. intended vs. unintended filter changes, medication adjustments, fluid management, small solute clearance, adverse events, catheter dysfunction) as proposed by Rewa et al.^16^ and others^12–14^. Finally, this work exemplifies the ability to nurture collaborative and quality improvement work in the ICU.

Our findings indicate that through developing a multidisciplinary CRRT team, standardizing CRRT protocols, integrating machine/EHR data, and reinforcing education, we were able to improve adherence to protocols, confidently and sustainably track CRRT delivery and reduce filter resource utilization at our institution. Additional studies are needed to confirm these results and the impact of these QI initiatives on processes of care and patient-centered outcomes.

## Methods

### Setting

The University of Kentucky (UK) Albert B. Chandler Hospital is a 945-bed acute care hospital with more than 37,000 admissions and approximately 2500 medical ICU admissions per year^23,24^. The Nephrology ICU consultation team (consisting of one attending, one fellow, and one resident or advanced practitioner) provides diagnostic and therapeutic services for patients with AKI and ESKD requiring CRRT in the ICU. The provision of CRRT leans on a multidisciplinary approach, including intensivists, ICU nurses, pharmacists and many other specialists. We perform CRRT for approximately 500 patients annually, corresponding to ~ 3200 CRRT patient-day per year.

This QI study describes the development, implementation and outcomes of a quality assurance system to support the provision of CRRT to adult patients in the ICU. This QI study was approved by the University of Kentucky Institutional Review Board (IRB) of the Office of Research Integrity (17-0444-P1G). Requirement for informed consent was waived by the IRB (https://www.research.uky.edu/office-research-integrity). All methods were carried out in accordance with relevant guidelines and regulations.

### Implementation

**Figure 3:**
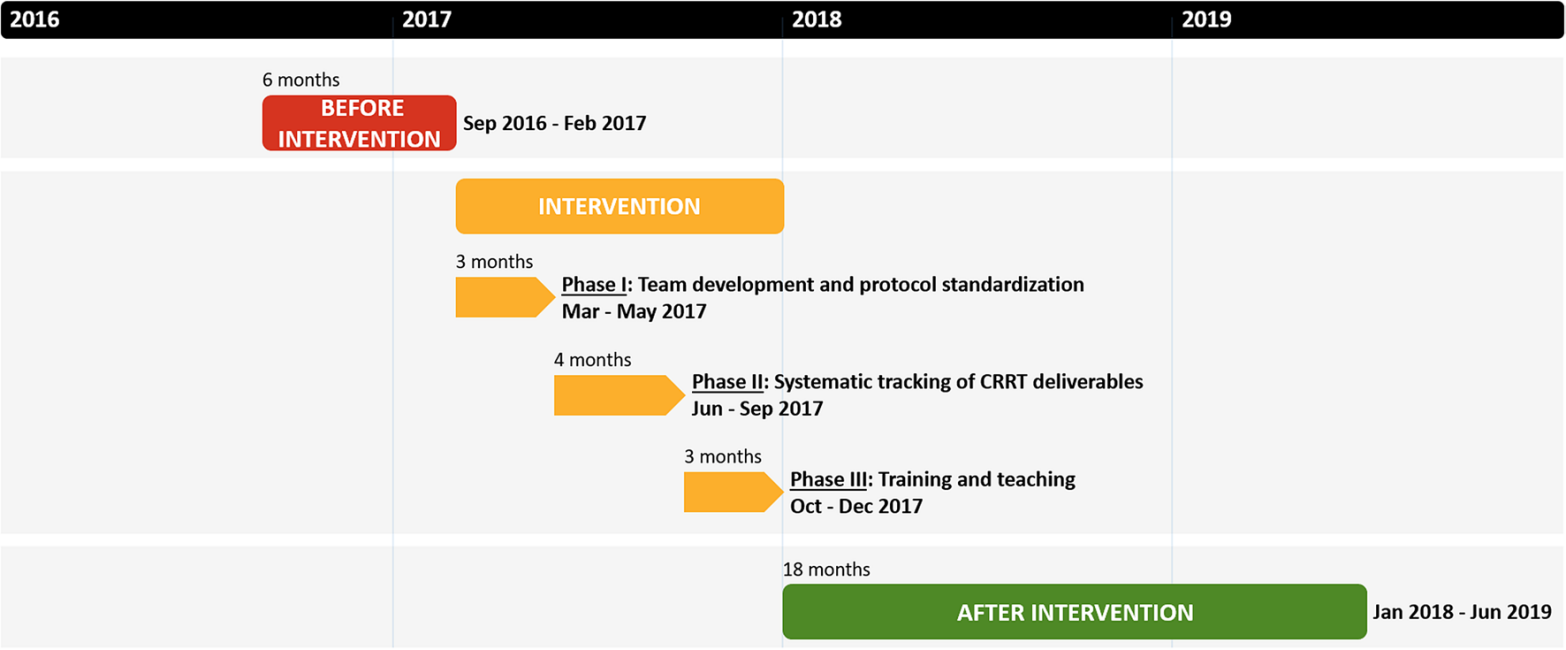
Study periods and phases of quality improvement interventions. *CRRT* continuous renal replacement therapy.

We developed and implemented a quality assurance system using a step-wise approach in 3 phases (intervention period, total of 10 months) (Fig. 3). The Phase I (3 months) consisted in the (a) assembly of a multidisciplinary team and the (b) standardization of the CRRT protocol tailoring institutional logistics and needs. The Phase II (4 months) involved the (c) creation of electronic CRRT flowsheets (Supplementary Fig. S2) and the (d) selection, monitoring and reporting of CRRT quality metrics. Finally, the Phase III (3 months) focused on the (e) enhancement of education to clinicians and ICU nurses.

We also established 3 study periods with the purpose of evaluating the impact of our intervention (Fig. 3). These periods were defined according to data availability before the intervention and the step-wise completion of milestones during the intervention phases. These periods consisted of: (1) before intervention (September 2016 to February 2017, 6 months); (2) intervention (March 2017 to December 2017, 10 months); and (3) after intervention (January 2018 to June 2019, 18 months).

### Intervention

The 10-month intervention was carried out from March 2017 to December 2017 in 3 step-wise phases described in Table 3. Team development and protocol standardization (Phase I) allowed the establishment of the multidisciplinary quality assurance team (Supplementary Fig. S1) which reached consensus for CRRT protocols (modality, dose, access, and anticoagulation) based on revision of current guidelines, evidence-based practices, institutional logistics and local expertise. For the selection of CRRT QI metrics to monitor CRRT delivery (Phase II), we adapted via consensus the quality control system proposed by Joannes-Boyau et al.^13^ including *structure* metrics encompassing the CRRT provider (e.g., specialized team, education program) and the CRRT prescription (e.g. modality, prescribed dose, anticoagulation); and *process* (performance) metrics (e.g., delivered dose, filter life span, access alarms). A total of 11 CRRT QI metrics (study outcomes) under the domains of *structure* and *process*, and 3 subdomains (provider, prescription and performance) in addition to economic savings specific to filter use were selected for this study and are described in detail in Table 4. Preconditions such as type of CRRT machine, filter and catheter were not changed throughout the study period. For training and teaching (Phase III), we conducted dynamic monthly assessments of learning needs to tailor teaching activities for ICU nurses (New and Super Users) and clinicians. This was done by auditing CRRT charting, assessing machine specific performance data, and making rounds in the ICUs (Super Users were available in both day and night shifts). Clinical duties were not compromised as the QI officer and leadership of the program have protected time for these activities. Furthermore, physicians, ICU nurses and other healthcare professionals were invited to attend bi-monthly QI meetings in order to voice all concerns they were experiencing as well as to conduct an iterative assessment of the program.

**Table 4:** Description of selected CRRT quality improvement metrics for this study.

**Quality domain: structure**	
**Provider**	
1. Specialized CRRT team	The number of multidisciplinary experts that constitutes the CRRT QI team
2. Education and training program	The number of education sessions for clinicians, ICU nurses and other healthcare professionals
**Prescription**	
3. CRRT modality	The percentage of the total CRRT treatments that used CVVHDF as the main modality established by protocol
4. Anticoagulation	The percentage of the total CRRT treatments that used RCA
5. Total RCA/RCA-CRRT hours	The average total RCA hours divided by total CRRT hours in patients that used RCA
**Quality domain: process**	
**Performance**	
6. Delivered effluent dose	The average delivered CRRT effluent flow rate (ml/kg/h)
7. Delivered vs. prescribed effluent dose	The average delivered CRRT effluent dose divided by prescribed dose
8. Filter life span	The average time (in hours) of individual filter utilization
9. Filters per patient	The average total number of filters used divided by the total number of patients on CRRT
10. CRRT access alarms	The average number of CRRT access alarms per treatment day, reflecting catheter malfunction (high venous pressure in return line or low arterial pressure in access line)
11. Economic savings	The average gross total filter cost per 100-patient receiving CRRT

*CRRT* continuous renal replacement therapy, *CVVHDF* continuous veno-venous hemodiafiltration, *ICU* intensive care unit, *QI* quality improvement, *RCA* regional citrate anticoagulation.

### Study data

Data from all adult patients (≥ 18 years old) receiving CRRT in the ICU during the study period were analyzed. Demographic and clinical data were collected by automated digital extraction from the EHRs through a flexible dashboard (Tableau, Supplementary Fig. S3), which allowed data to be downloaded as spreadsheets or graphics for review and further analysis. Data extraction was validated through individual review of EHRs. Performance data from CRRT machines were extracted from accessing individual machine data cards during the study period.

### Statistical analysis

Categorical variables are expressed as counts and percentages. Continuous variables are reported as mean and standard deviation (SD), or median and interquartile range (IQR) according to data distribution. A chi-square test or Fisher’s exact test was used to compare categorical variables when appropriate. Continuous variables with a normal distribution were compared using an independent Student t-test; in contrast, variables not exhibiting a normal distribution were compared using the Mann–Whitney U test. All statistical analyses were performed using SPSS 24.0 (IBM SPSS Statistics for Windows, Version 24.0. Armonk, NY: IBM Corp). A two-tailed p < 0.05 was considered statistically significant.

### Ethics approval and consent to participate

This QI study was approved by the University of Kentucky Institutional Review Board (IRB) of the Office of Research Integrity (17-0444-P1G). Requirement for informed consent was waived by the IRB (https://www.research.uky.edu/office-research-integrity). All methods were carried out in accordance with relevant guidelines and regulations.

## Supplementary information

Supplementary Information.

## Data Availability

The datasets used and/or analyzed during the current study are available from the corresponding author on reasonable request.
