# Supplementary material for: Development, implementation and outcomes of a quality assurance system for the provision of continuous renal replacement therapy in the intensive care unit
Source: Sci Rep. 2020 Nov 26;10:20616. doi: 10.1038/s41598-020-76785-w (PMC7692557; doi:10.1038/s41598-020-76785-w)

Development, Implementation and Outcomes of a Quality Assurance System for the Provision of Continuous Renal Replacement Therapy in the Intensive Care Unit

Eloy F. Ruiz, MD[1]; Victor M. Ortiz-Soriano, MD[1]; Monica Talbott, BSN, RN[1]; Bryan A. Klein, BSN, RN, CCRN[1]; Melissa L. Thompson Bastin, PharmD, BS, BCPS[2]; Kirby P. Mayer, DPT, PhD[3]; Emily B. Price, MBA[1]; Robert Dorfman, BSN, RN, CCRN[1]; Brandi N. Adams, MSN, MHA, CDN, RN PCM Dialysis[1]; Lisa Fryman, DNP, RN[1]; Javier A. Neyra, MD, MSCS[1*];

on behalf of the University of Kentucky CRRT Quality Assurance Group

1. Division of Nephrology, Bone and Mineral Metabolism, Department of Internal Medicine, University of Kentucky, Lexington, KY, USA.
2. College of Pharmacy, Department of Pharmacy Practice and Science, University of Kentucky, Lexington, KY, USA.
3. Department of Physical Therapy, University of Kentucky, Lexington, KY, USA.

Figure S1: Structure of the multidisciplinary CRRT Quality Assurance team.

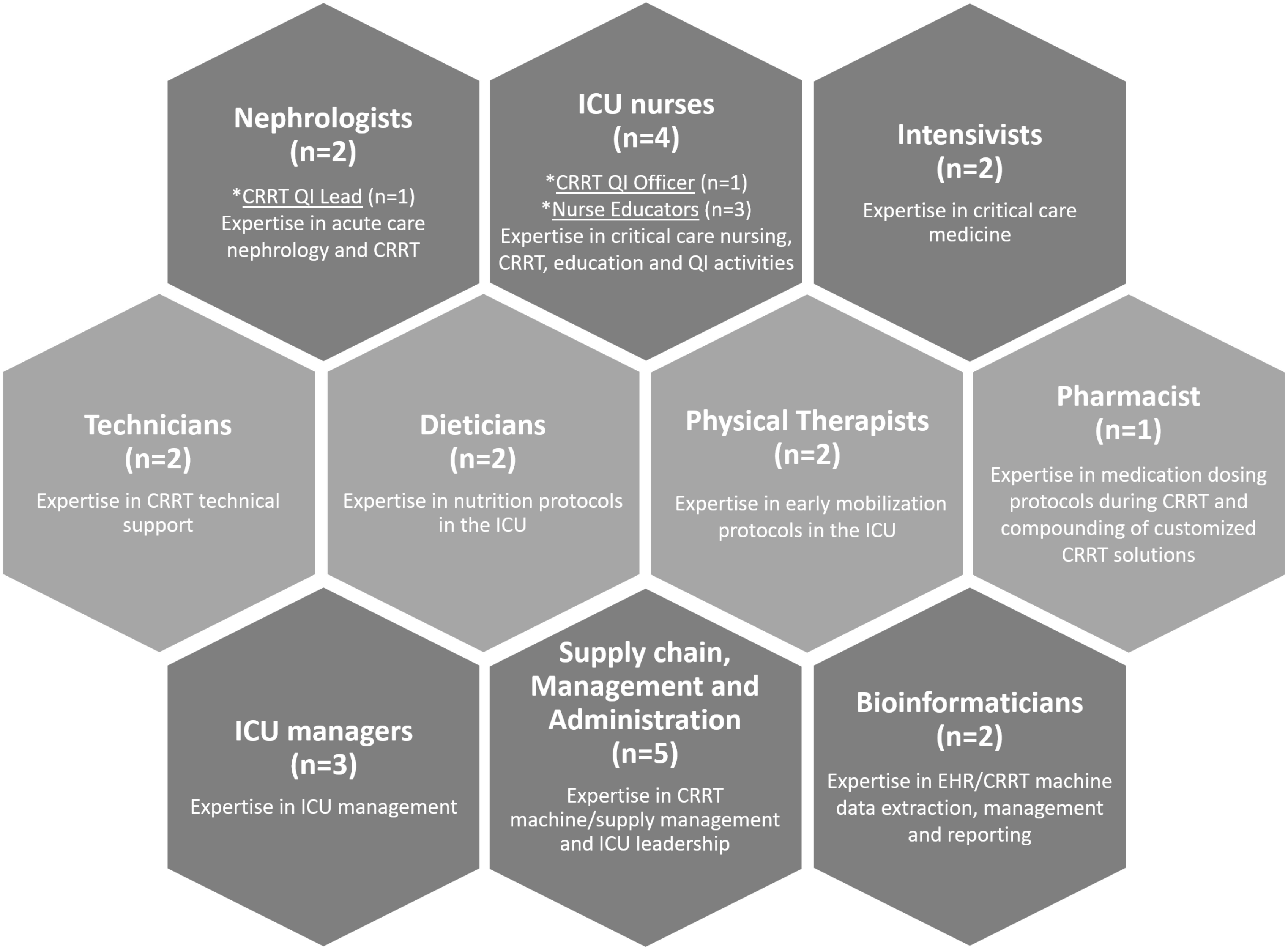

Abbreviations: **CRRT**, continuous renal replacement therapy; **EHR**, electronic health record; **ICU**, intensive care unit; **QI**, quality improvement.

Figure S2: Sample of electronic CRRT flowsheet.

	15-Aug-2018 22:00	15-Aug-2018 23:00	16-Aug-2018 0:00	16-Aug-2018 1:00
**CRRT Fluid Rates**				
Rates--per policy chart q 1 hour				
PBP (white), ml/hr	300	300	300	300
Dialysate (green), ml/hr	2000	2000	2000	2000
Post-filter replacement (Purple), ml/hr	1000	1000	1000	1000
Calcium Chloride, ml/hr (systemic)	30	30	40	40
**CRRT Fluid Solutions**				
Solutions				
PBP (White)	Citrate	Citrate	Citrate	Citrate
Dialysate (Green)	Phoxillum B22K 4/0	Phoxillum B22K 4/0	Phoxillum B22K 4/0	Phoxillum B22K 4/0
Post-filter replacement (purple)	Phoxillum B22K 4/0	Phoxillum B22K 4/0	Phoxillum B22K 4/0	Phoxillum B22K 4/0
**Fluid Balance**				
Balances				
1. Hourly UF goal (MD Order)	Net negative	Net negative	Net negative	Net negative
2. Net negative value (MUST BE A NEGATIVE NUMBER!!)	-50	-50	-50	-50
3. Perform Calculations Now				
Patient Intake	53.5	64.4	90.2	58.9
Patient Out	10	6	20	35
Patient Balance	43.5	58.4	70.2	23.9
Deficit From Prior Hour	0	-27.5	-57.1	-78.9
Actual fluid removal (Prismaflex)	127	121	138	142
Actual Balance	43.5	30.9	13.1	-55
Suggested Fluid Removal Next Hour	93.5	80.9	63.1	-5
Machine Number				
**Prismaflex Parameters**				
Parameters				
Mode	CVVHDF	CVVHDF	CVVHDF	CVVHDF
BFR ml/min	200	200	200	200
Access	-53	-57	-52	-56
Filter	131	132	135	127
Effluent	24	15	21	14
Return	73	74	78	68
Pressure Drop	53	26	29	41
Tmp	47	75	73	62
Filter Fraction (%)	16	17	17	17
Deaeration Chamber Q 1 hr	Yes	Yes	Yes	Yes

Abbreviations: **BFR**, blood flow rate; **CRRT**, continuous renal replacement therapy; **CVVHDF**, continuous veno-venous hemodiafiltration; **PBP**, pre-blood pump; **Tmp**, transmembrane pressure; **UF**, ultrafiltration.

Figure S3: Screenshots of Tableau tool for tracking CRRT clinical data.

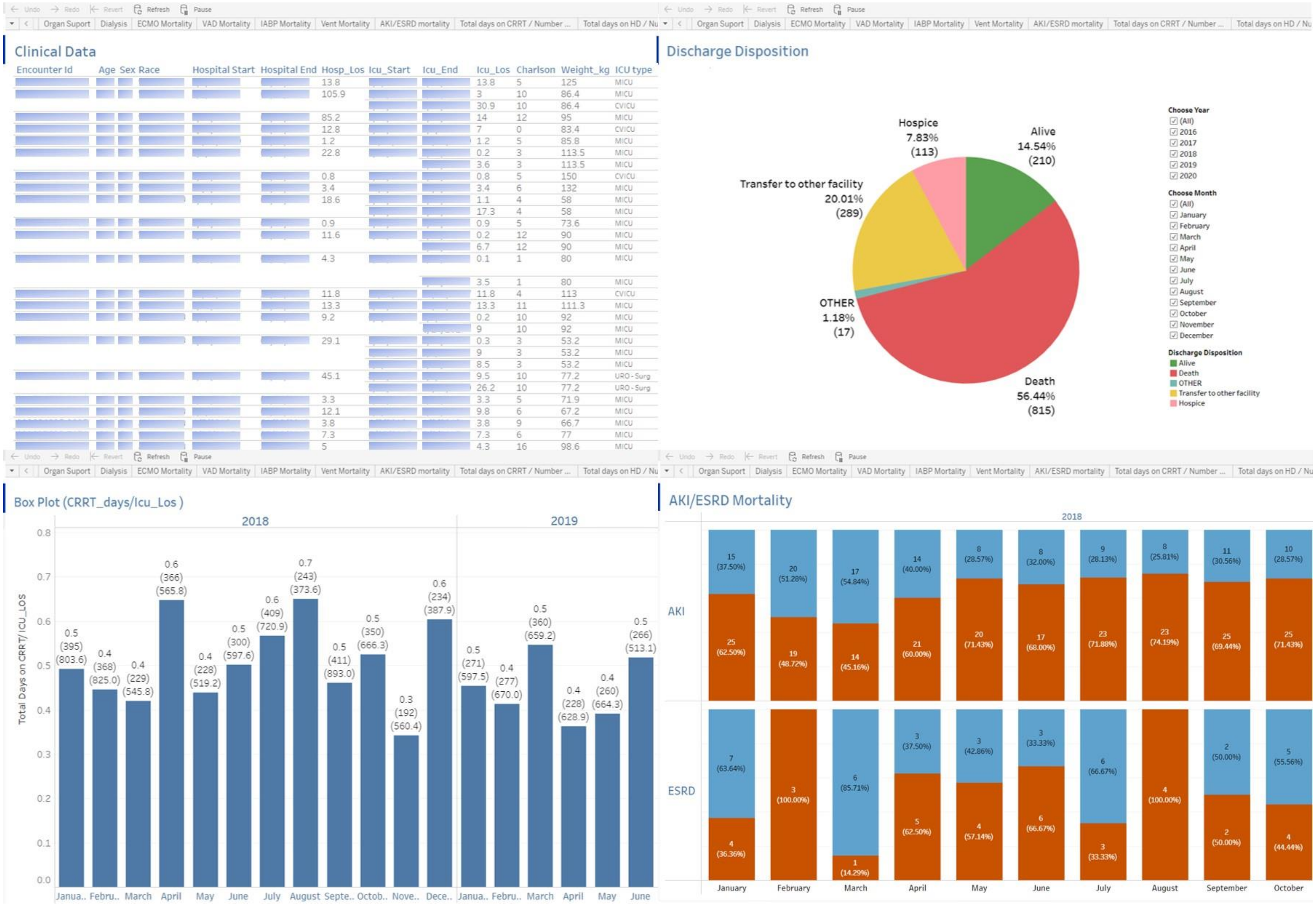

Supplement: Supplementary file 1 — Supplementary Information. [file 41598_2020_76785_MOESM1_ESM.pdf]
